# Hydralazine-Induced Lupus Presenting With Pancytopenia: A Case Report

**DOI:** 10.7759/cureus.29793

**Published:** 2022-09-30

**Authors:** Yudhveer Brar, Nhia R Yang, Anupama Poliyedath

**Affiliations:** 1 Internal Medicine, Saint Agnes Medical Center, Fresno, USA

**Keywords:** hydralazine-induced pancytopenia, left-sided pleural effusion, sle, pericarditis, drug-induced lupus (dil)

## Abstract

Hydralazine-induced lupus leading to pancytopenia is an uncommon presentation and can have systemic effects on the body. We present the case of a 73-year-old male with complaints of fever, night sweats, and non-intentional weight loss. Complete blood count pathology review showed pancytopenia with no blasts. Detailed infectious disease workup, including Coccidioidomycosis and tuberculosis, was negative. Rheumatological workup including rheumatic factor, anti-smith, anti-double-stranded DNA antibodies, and anti-ribonuclear proteins, was negative. Anti-nuclear antibodies and anti-histone antibodies were found to be positive. This led to the diagnosis of hydralazine-induced lupus. Hence, hydralazine was immediately discontinued which led to rapid improvement.

## Introduction

Hydralazine was implemented into daily use to treat hypertension in 1951. This was an era during which the only alternatives were ganglionic blockers, such as hexamethonium. Patients started noticing presentations that were similar to intrinsic lupus erythematosus or rheumatoid arthritis the following year [1]. Hydralazine leading to lupus-like symptoms has an incidence rate of approximately 7% and most commonly presents with fever, myalgias, fatigue, rash, serositis, arthritis, pleural effusion, fever, hepatosplenomegaly, pericarditis, glomerulonephritis, and neuropsychiatric symptoms [1].

## Case presentation

A 73-year-old male with a medical history of basal cell skin carcinoma, hyperlipidemia, type 2 diabetes mellitus, and hypertension presented to the clinic with chief complaints of fever, night sweats, and unintentional weight loss. The patient had been on hydralazine 50 mg three times daily for the past three years.

The patient had a history of recurring fever, primarily in the evenings, since January 2020 and had been dealing with these symptoms for the past five months. He mentioned that fevers occurred two to three times per week, with temperatures ranging from 99°F to 102°F. He endorsed night sweats that occurred approximately two to three times per week. This led to him waking up at night with clothes and hair drenched in sweat. He took Advil to relieve his fever. The patient had a history of unintentional weight loss and had lost 15 pounds in the past five months. He denied any changes in appetite, fatigue, blood in the stool, or dark-colored stools. There was no history of travel outside the United States. A physical examination demonstrated blood pressure of 180/90 mmHg and a heart rate of 120 beats per minute. No cardiac murmurs, rubs, or gallops were appreciated on examination. Lungs revealed faint right-sided crackles. There was no jugular venous distention. The patient endorsed recent unusual hair loss. No eyrthema nodusum, erythematous papules, or purpura were noted. Hepatomegaly or splenomegaly was not palpated on examination. The rest of the examination revealed no abnormalities. Laboratory findings are presented in Table 1.

**Table 1:** Laboratory findings consistent with the diagnosis of hydralazine-induced lupus. WBC: white blood cells; CRP: C-reactive protein; TB: tuberculosis

Parameters	Results	Normal range
WBC (m/mm^3^)	4	4.5–11
Platelets (m/mm^3^)	83	150,000–450,000
Hemoglobin (g/dL)	9.9	13.2–16.6
CRP (mg/L)	15.3	Less than 10
Antinuclear antibody titers	1:1280	Titers less than or equal to 1:40
Anti-histone antibody	1.6	Less than 1 unit
Anti-double-stranded DNA	Negative	
Anti-smith	Negative	
Rheumatoid factor	Negative	
Anti-ribonuclear protein	Negative	
Nucleosomal antibody	Positive	
Coccioidal serology	Negative	
Quantiferon TB	Negative	

**Figure 1:**
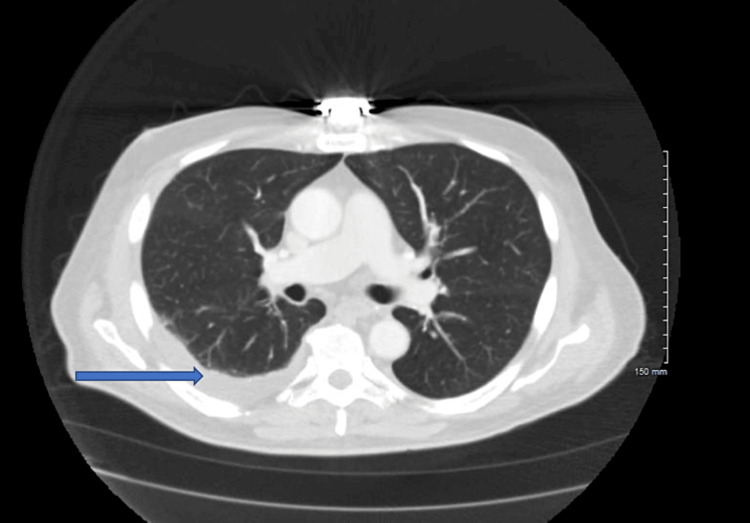
Right-sided pleural effusion secondary to hydralazine.

Complete blood count pathology review showed pancytopenia with no blasts. Abdominal and pelvis computed tomography with contrast showed moderate right pleural effusion (Figure 1). Ultrasound-guided thoracentesis showed reactive mesothelial cells, lymphocytes, and macrophages. No malignancy was noted. Immunofixation electrophoresis urine was negative for paraproteins.

Eventually, the decision was made to discontinue hydralazine as it was the most likely culprit. The patient had a remarkable improvement in symptoms after the discontinuation of hydralazine. On a follow-up appointment after two months of discontinuation of hydralazine, he revealed that his symptoms of night sweats, fevers, and weight loss had resolved.

## Discussion

In patients who are diagnosed with hydralazine-induced lupus, human leukocyte antigen (HLA)-DRw4 is present in 73% of patients [2]. Hydralazine is primarily metabolized by acetylation in the liver. Enzyme activity rates differ in fast and slow acetylators [2]. Faster elimination occurs in patients with faster acetylation of the drug, which is protective against reactive metabolites [2]. In a study of 57 patients diagnosed with hypertension receiving hydralazine, 22 of the 33 slow acetylators showed the development of antinuclear antibodies (ANAs) compared to only nine of the 24 fast acetylators. Of the patients who presented with lupus-like symptoms, 12 belonged entirely to the group of slow acetylators [2]. Even though most drug-induced systemic lupus erythematosus (SLE) cases are common among slow acetylators, there have been rare instances where rapid acetylators have been diagnosed with drug-induced SLE [2].

Early detection of SLE is important, and patients should be evaluated to detect early symptoms of the syndrome at each patient visit. Before initiating hydralazine therapy, it is imperative to get a baseline ANA test [2]. It is also to be noted that routine follow-up ANA tests are neither required nor recommended as a positive test does not indicate drug-induced SLE. Overall, 50% of the patients on hydralazine therapy test positive for ANA but do not have SLE [2].

Patients who endorse common symptoms such as fever, weight loss, and musculoskeletal symptoms while being on hydralazine should be closely monitored. ANA levels as well as anti-histone antibodies and detailed lab work should be ordered to rule out drug-induced SLE [3]. After the publication of the African American heart failure trial in 2004, hydralazine in combination with isosorbide dinitrate became heavily used among African American patients [4]. Due to the widespread use of hydralazine, physicians should be vigilant regarding the side effects of hydralazine. Hydralazine should be immediately discontinued if any of the above symptoms are reported.

## Conclusions

In our case report, our patient continued to endorse symptoms for five months. During these five months, he pursued “doctor shopping” as his symptoms were not being relieved. He finally presented to our residency clinic looking for answers. After reviewing his medications in detail and ruling out the necessary infectious etiology, hydralazine was found to be the most likely culprit. Discontinuing hydralazine led to rapid resolution of the patient’s symptoms. Hence, it is imperative for physicians to closely monitor medication lists as most of the time patients are dealing with medication side effects. In our case, it was hydralazine inducing lupus-like symptoms.
